# Supplementary figures and images for: Modelling the spatial dynamics of oncolytic virotherapy in the presence of virus-resistant tumour cells
Source: PLoS Comput Biol. 2022 Dec 6;18(12):e1010076. doi: 10.1371/journal.pcbi.1010076 (PMC9767357; doi:10.1371/journal.pcbi.1010076)

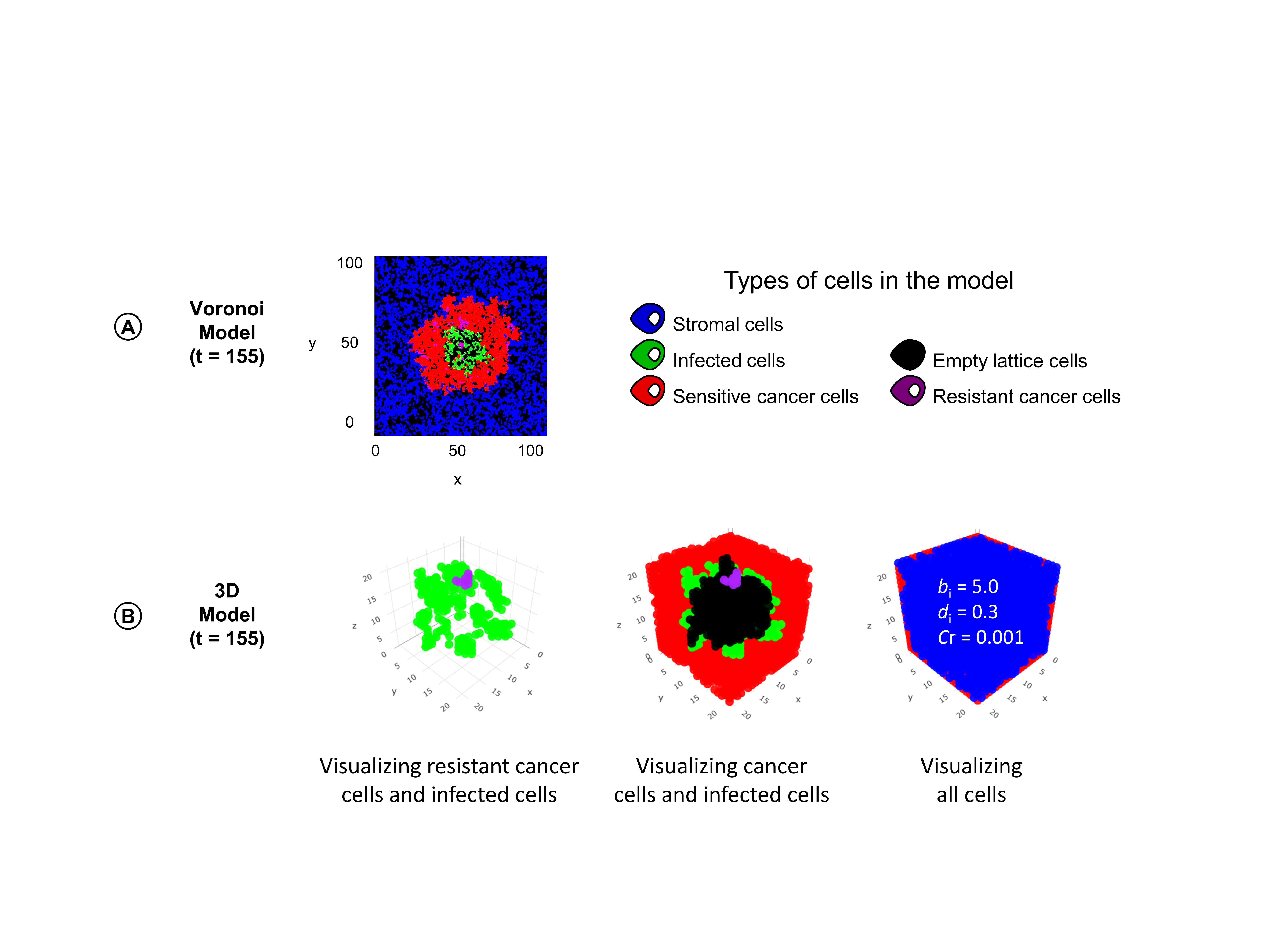

Supplement: S1 Fig — A snapshot of the (A) Voronoi model and the (B) 3D model at a runtime (t = 155) was taken for comparison of the model dynamics. The grid size was set at 1002 for the Voronoi model and at 223 for the 3D model to have a comparable (~10,000) number of total lattice cells. The parameter values of rate of viral spread (bi), death rate of infected cells (di) and probability of becoming resistant (Cr) is indicated in the figure. (TIF) [file pcbi.1010076.s001.TIF]

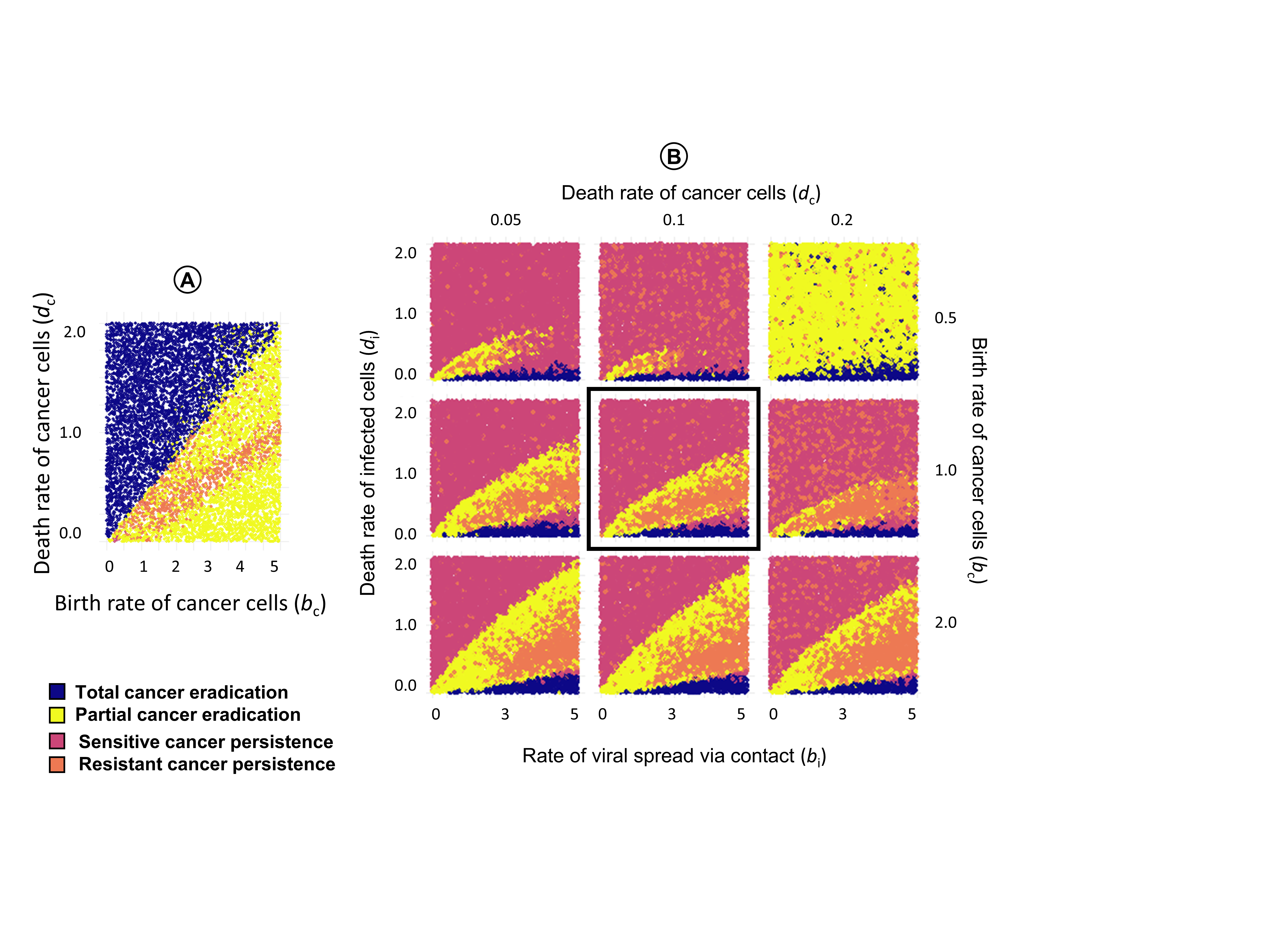

Supplement: S2 Fig — Therapeutic outcomes in relation to the birth (bc) and death (dc) rates of cancer cells for the Voronoi model were considered by keeping the rate of viral spread (bi) and infected cell death rate (di) at their default values (A) or in a range (B). 10,000 simulations were run for each panel, and each point corresponds to one simulation. With the exception of the investigated parameters, all parameters were at their default values. (TIF) [file pcbi.1010076.s002.TIF]

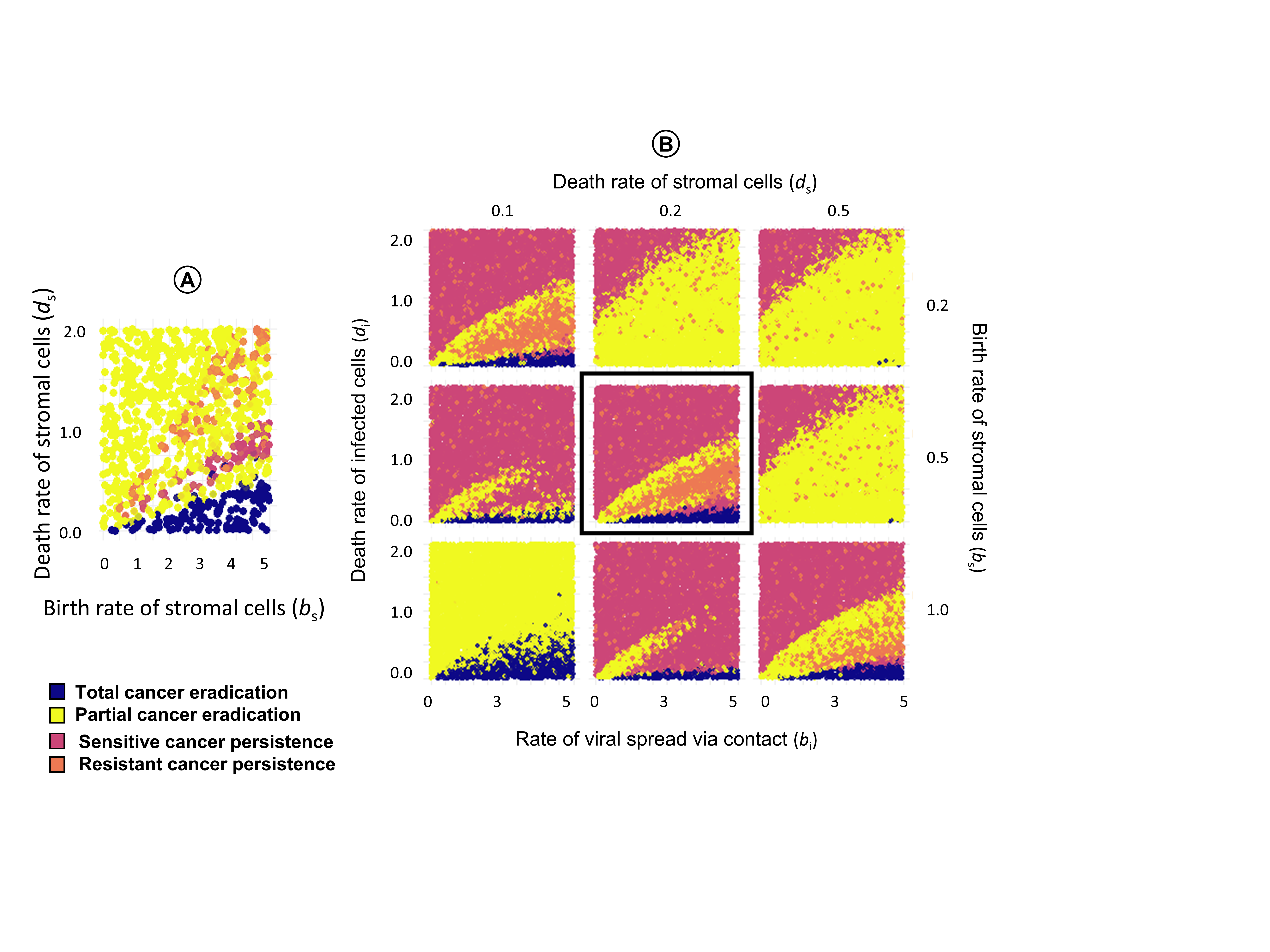

Supplement: S3 Fig — Therapeutic outcomes in relation to the birth (bs) and death rates of stromal cells (bs) for the Voronoi model were considered by keeping the rates of viral spread (bi) and death (di) at their default values (A) or in a range (B). 10,000 simulations were run for each panel, and each point corresponds to one simulation. With the exception of the investigated parameters, all parameters were at their default values. (TIF) [file pcbi.1010076.s003.TIF]

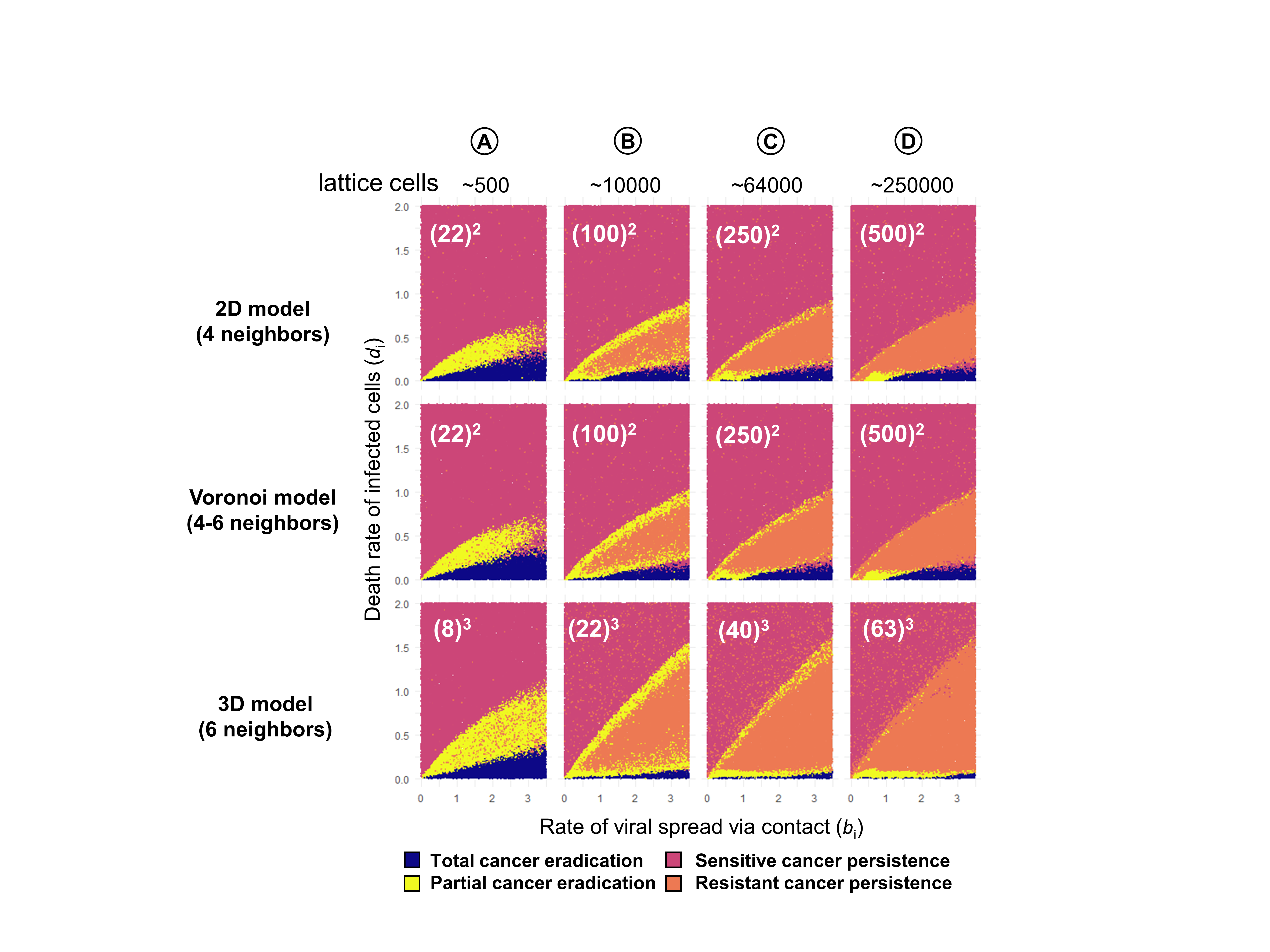

Supplement: S4 Fig — Therapeutic outcomes in relation to the rates of viral spread via contact (bi) and death rate of infected cells (di) for the three spatial configurations considered (regular 2D grid, 2D Voronoi model, regular 3D grid) and for various population sizes (A) about 500 lattice cells; (B) about 10,000 lattice cells; (C) about 64,000 lattice cells; (D) about 250,000 lattice cells. The white text in each panel indicates how the population size relates to the grid dimensions. 50,000 simulations were run for each panel, and each point corresponds to one simulation. With the exception of population size, all parameters were at their default values. (TIF) [file pcbi.1010076.s004.TIF]

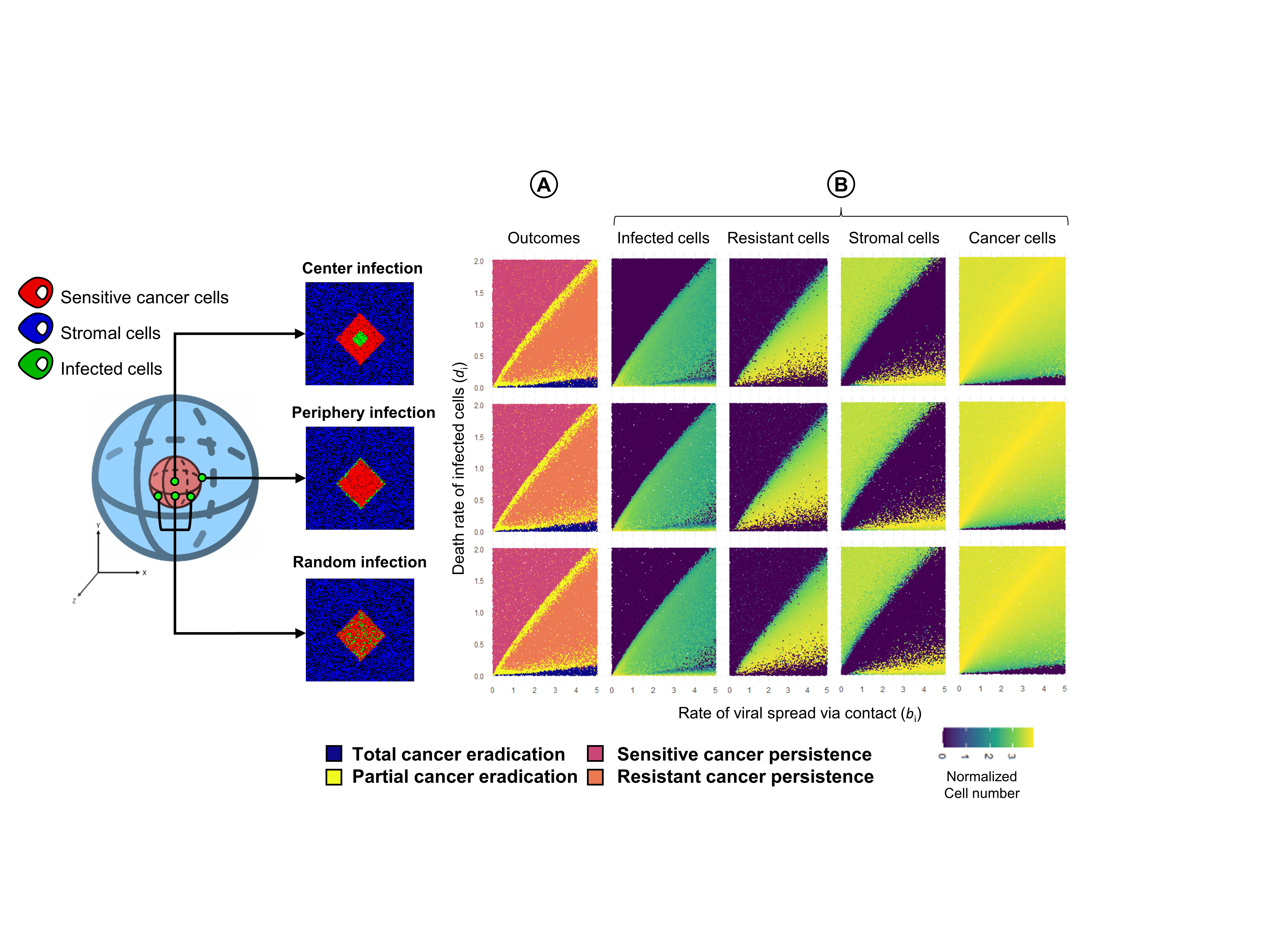

Supplement: S5 Fig — Simulation outcome in relation to the rates of viral spread via contact (bi) and death rate of infected cells (di) in the 3D model for three different forms of viral infection. As indicated in the illustration on the left, virus infection in the tumour is initiated either in the centre (top row), or from the periphery (middle row), or in a random manner (bottom row). For each infection scenario 50,000 simulations were run, which were classified according to (A) their therapeutic outcomes; and (B) the number of the different types of cells at the end of the simulations. All parameters were at their default values. The colour code is based on the logarithm of cell numbers. (TIF) [file pcbi.1010076.s005.TIF]

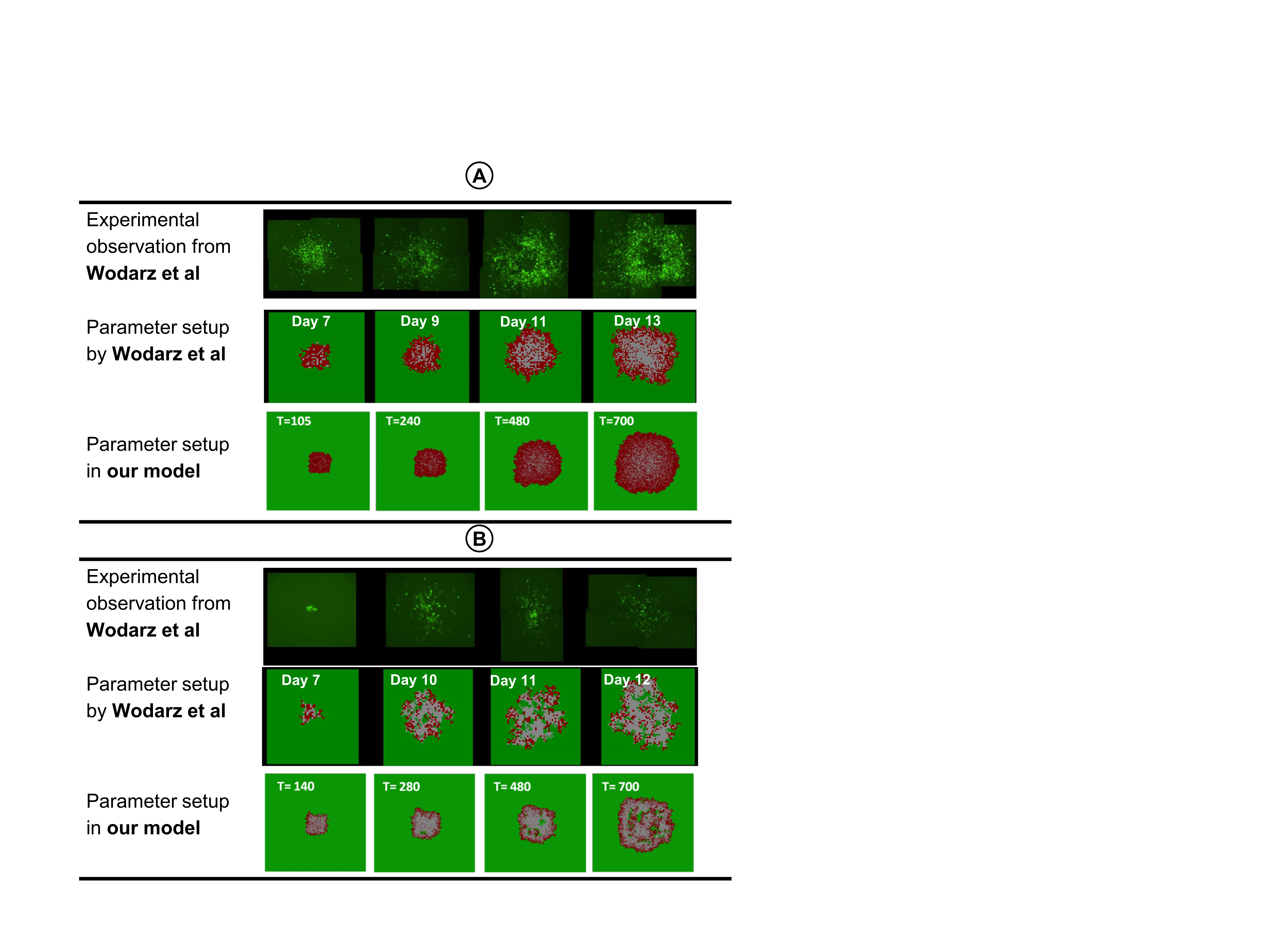

Supplement: S6 Fig — In their Figs 4 and 5, Wodarz and colleagues [14] compare the spatial dynamics of experimentally induced viral infections with the predictions of an agent-based model. Here, we illustrate that our event-based model creates similar spatial patterns as the discrete-time Wodarz model. (A) In both models, virotherapy leads to the radial spread of the virus and the extinction of cancer cells if the viral infection rate exceeds the death rate of infected cells by a factor of at least 10. In the simulation shown, bi = 0.1 and di = 0.001. (B) In both models, virotherapy results in the long-term coexistence of infected and uninfected cancer cells if the viral infection rate does neither exceed the death rate of infected cells nor the birth rate of uninfected cells by a factor of 10. In the simulation shown, bi = 0.1, bc = 1.0 and di = 0.01. In the experimental observations of Wodarz and colleagues, infected cells are labelled as green and non-infected cells as black. Regarding the simulations, we follow the conventions of Wodarz and colleagues to label infected cancer cells as red, non-infected cells as green, and empty space as white. (TIF) [file pcbi.1010076.s006.TIF]

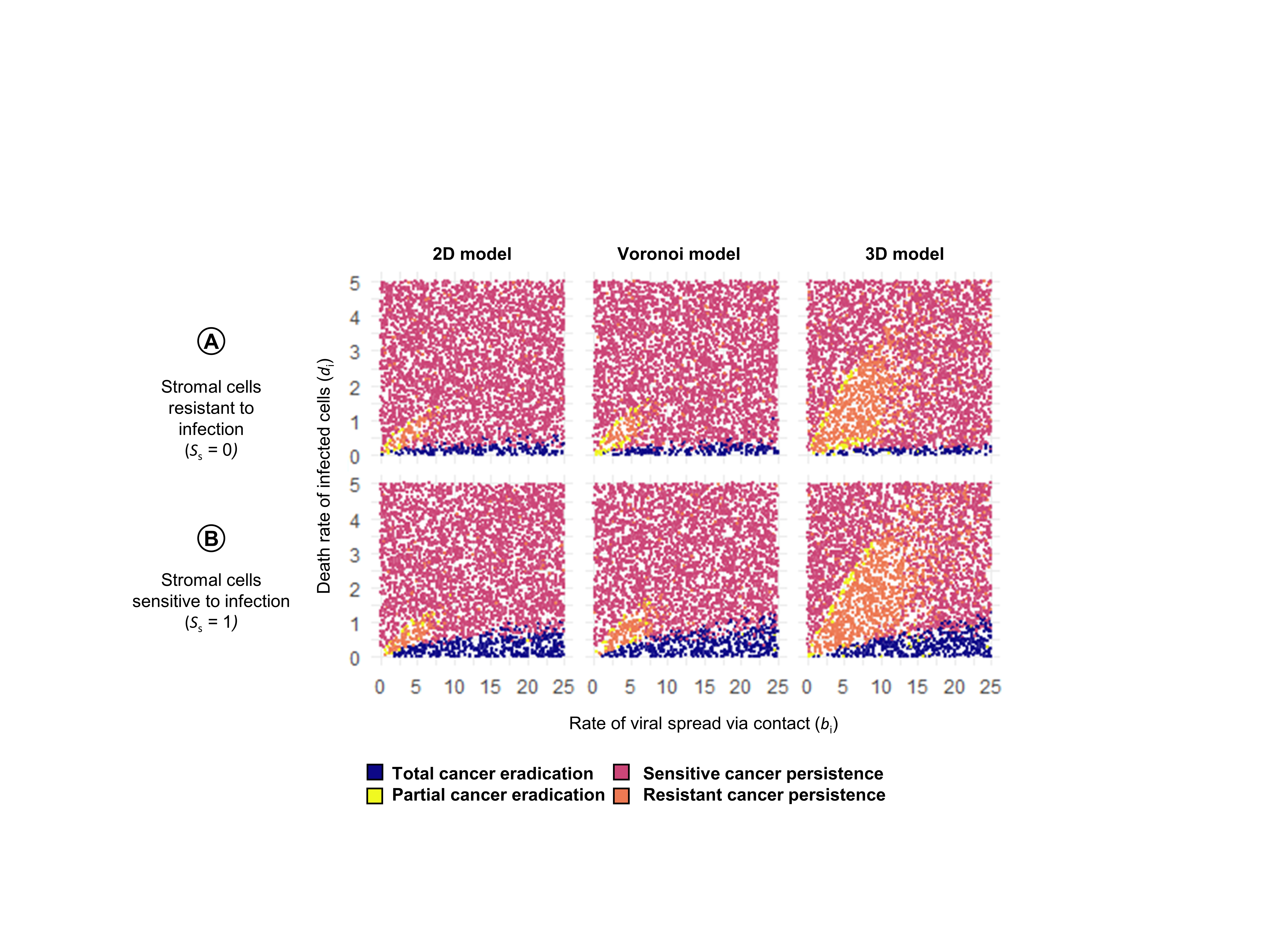

Supplement: S7 Fig — In the graphs of the main text (Fig 3), the rate of virus spread (bi) ranges from 0 to 5, while the death rate of infected cells (di) is from 0 to 2. For the three spatial configurations considered (regular 2D grid, 2D Voronoi model, regular 3D grid), the panels show the therapeutic outcome for a wider range of parameters, for two scenarios: (A) stromal cells cannot be infected by the virus; (B) stromal cells are sensitive to infection. Each panel in the figure is based on at least 10,000 simulations and each point represents one simulation. All parameters that are not varied are at their default values. (TIF) [file pcbi.1010076.s007.TIF]

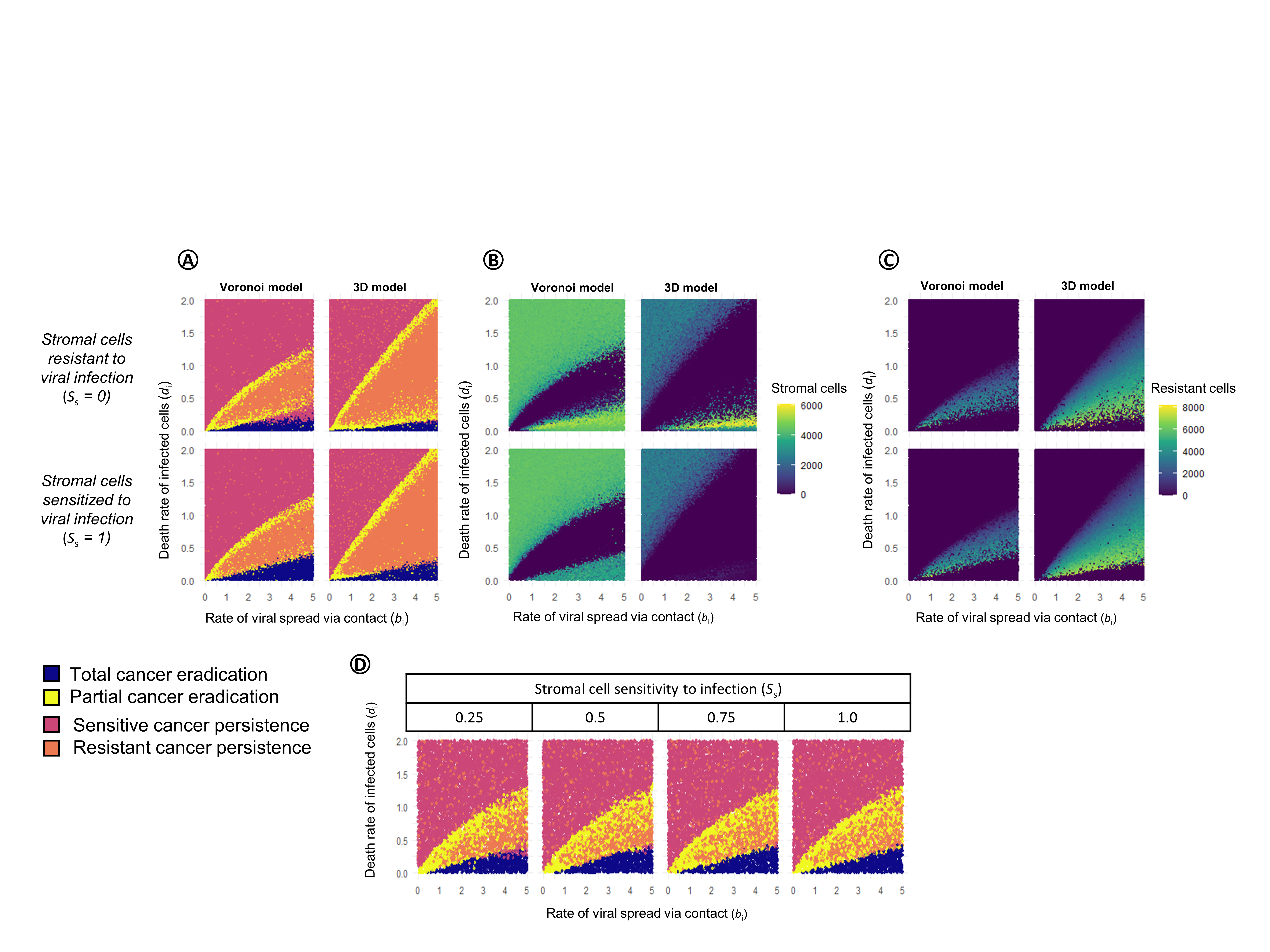

Supplement: S8 Fig — (A) Therapeutic outcome in relation to the rates of viral spread via contact (bi) and death rate of infected cells (di) for two spatial configurations (2D Voronoi model, regular 3D grid) and two different assumptions on stromal cells: stromal cells cannot be infected by the virus (top row); or stromal cells are sensitive to infection (bottom row). Each of the four panels represents 100,000 simulations. For the simulations in (A), the four panels indicate the number of stromal cells (B) and the number of resistant cancer cells (C) at the end of the simulation. The colour code is based on the absolute of cell numbers. (D) Therapeutic outcomes in the Voronoi model in relation to the rates of viral spread via contact and death rate of infected cells for different degrees of stromal cell susceptibility to viral infection. Each of the four panels represents 10,000 simulations. (TIF) [file pcbi.1010076.s008.TIF]

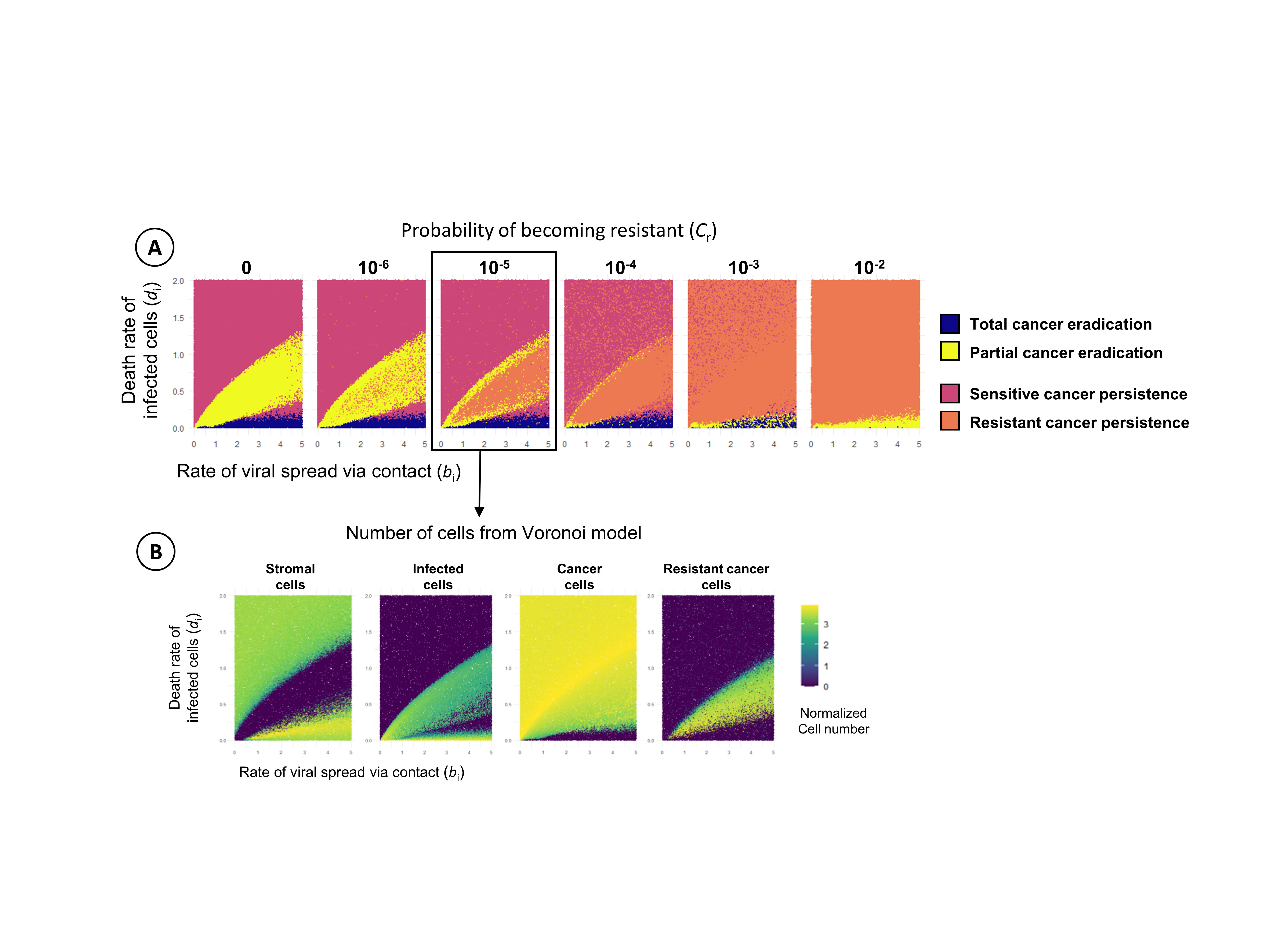

Supplement: S9 Fig — (A) Therapeutic outcomes in the 2D Voronoi model in relation to the rates of viral spread via contact (bi) and death rate of infected cells (di) for six probabilities of becoming resistant (Cr ranging from 0 to 10−2 per cell division). Each panel represents 100,000 simulations, and each point corresponds to one simulation. (B) Numbers of different types of cells at the end of the simulation for the default value (Cr = 10−5 per cell division). The colour code is based on the logarithm of cell numbers. (TIF) [file pcbi.1010076.s009.TIF]

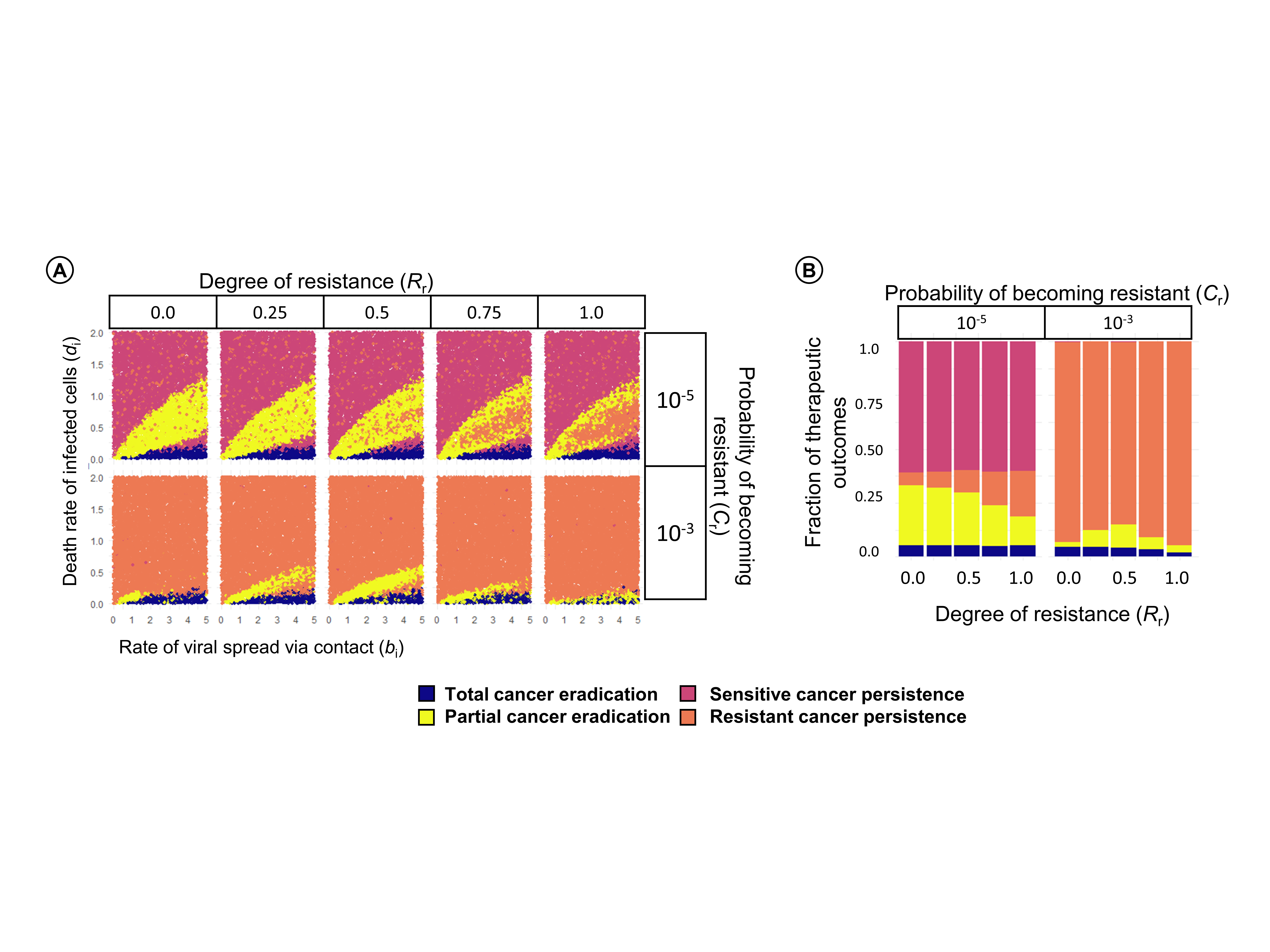

Supplement: S10 Fig — (A) Therapeutic outcomes in the 2D Voronoi model in relation to the rates of viral spread via contact (bi) and death rate of infected cells (di) for five degrees of resistance of cancer cells (Rr ranging from 0 to 1) and two production rates (Cr is 10−5 or 10−3 per cell division) of resistant cells. Each panel represents 10,000 simulations, and each point corresponds to one simulation. (B) The bar chart indicates the likelihood of the four outcomes for five values of degree of resistance (Rr) and the size of the four bars in the bar chart is proportional to the areas indicated by blue, yellow, red, and orange in (A). The bar chart for the production rate of resistant cells at 10−5 per cell division in (B) is the same as depicted in Fig 4B. (TIF) [file pcbi.1010076.s010.TIF]

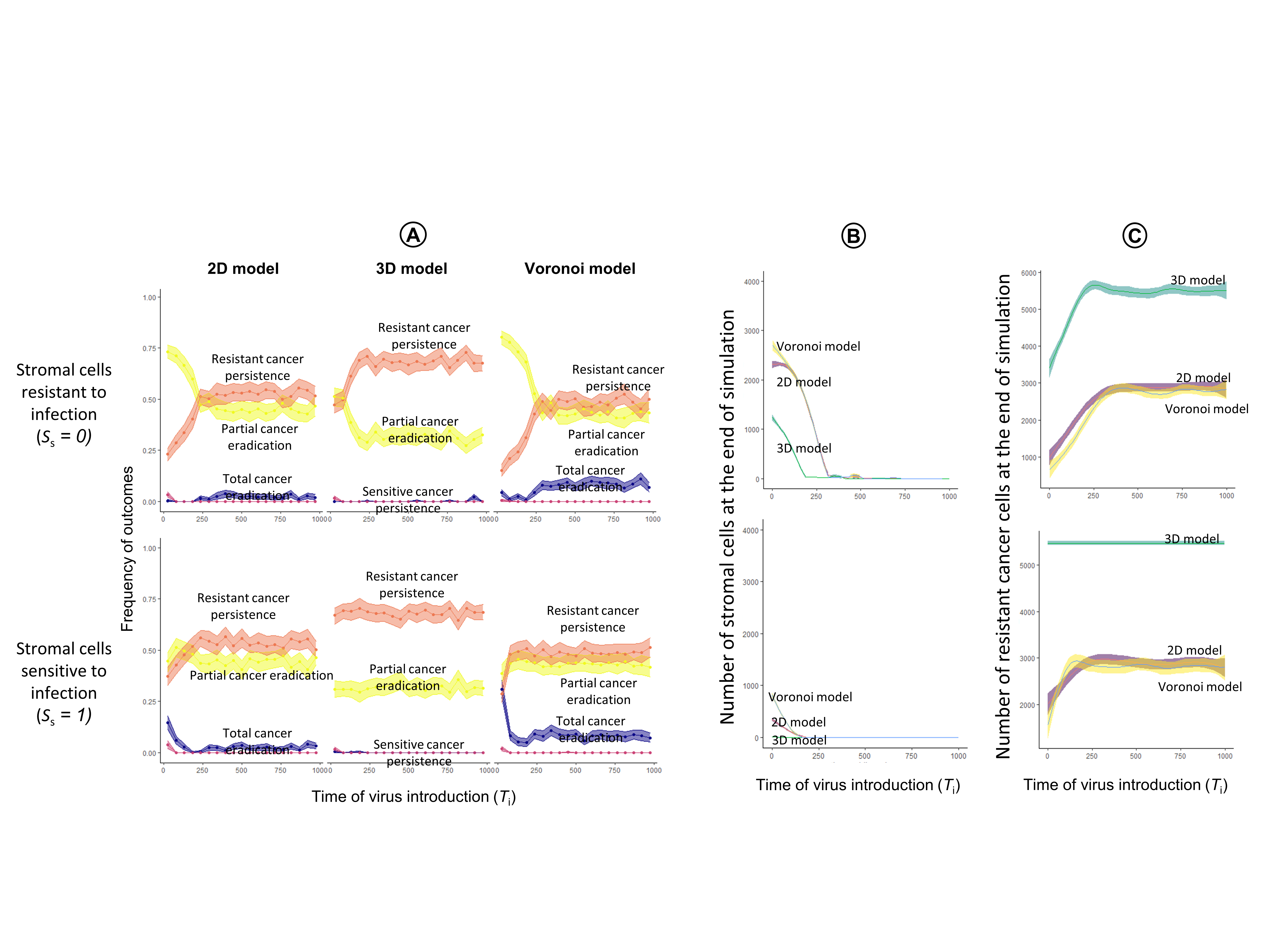

Supplement: S11 Fig — This figure corresponds to Fig 5 in the main text, which illustrates the effect of start of viral treatment (Ti) on the therapeutic outcome in the 2D Voronoi model. Here, the corresponding outcomes are shown in (A) for the regular 2D grid and 3D grid models. Effect of start of virotherapy (Ti) on the (B) number of stromal cells and (C) number of infection-resistant cancer cells at the end of the simulation is provided. Two scenarios are considered: stromal cells are resistant (top row) or sensitive (bottom row) to infection. The number of simulations per panel and the model parameters are as in Fig 5. (TIF) [file pcbi.1010076.s011.TIF]

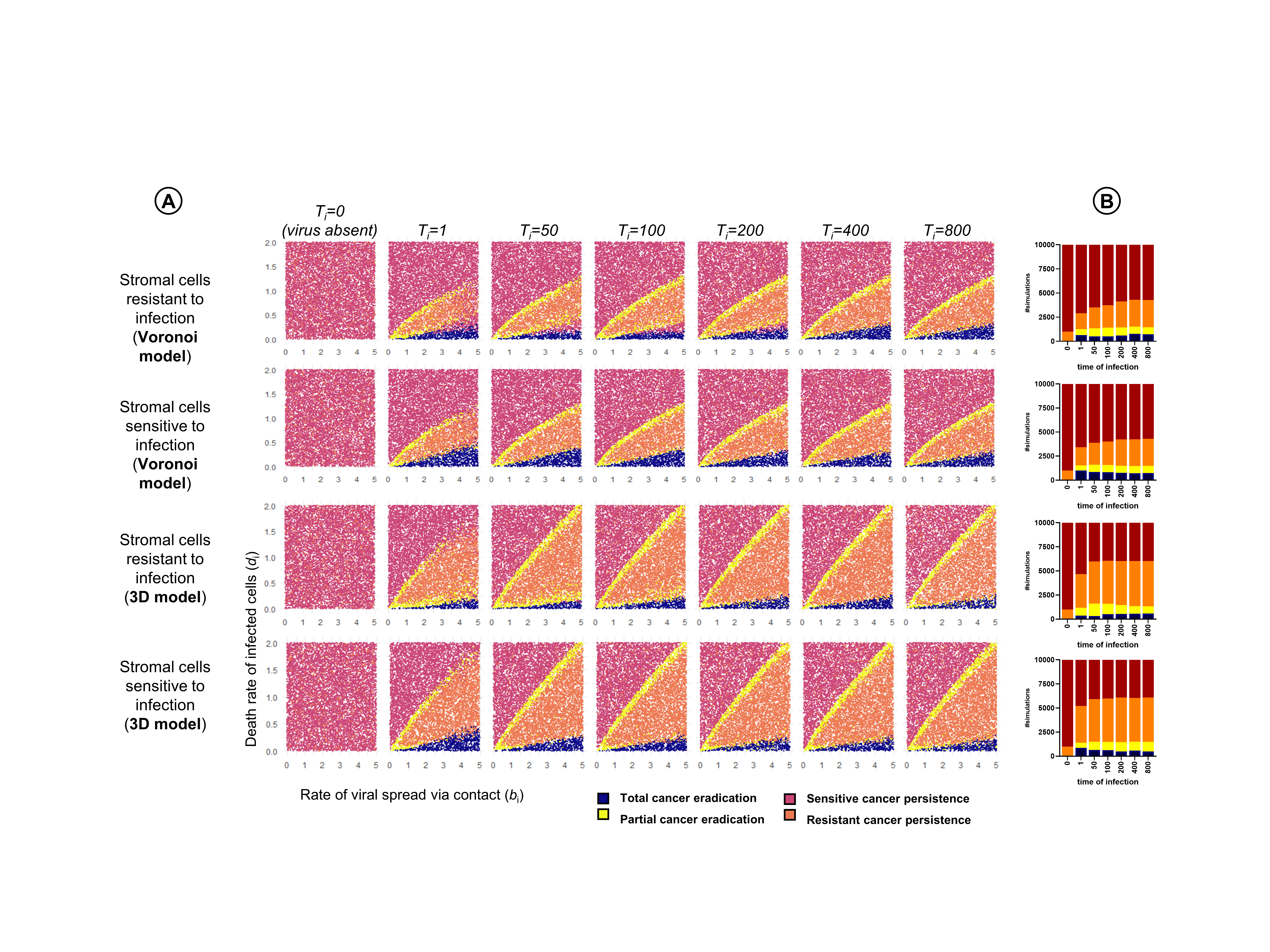

Supplement: S12 Fig — (A) Effect of the time of treatment (Ti) on the therapeutic outcome in the Voronoi model and 3D model. For the same range of parameter values as in Fig 3 (rate of viral spread (bi) and death rate of infected cells(di)) 10,000 simulations were run and classified as to their therapeutic outcome. (B) The bar chart indicates the likelihood of the four outcomes for different values of Ti. (TIF) [file pcbi.1010076.s012.TIF]
