# Supplementary material for: Modelling the spatial dynamics of oncolytic virotherapy in the presence of virus-resistant tumour cells
Source: PLoS Comput Biol. 2022 Dec 6;18(12):e1010076. doi: 10.1371/journal.pcbi.1010076 (PMC9767357; doi:10.1371/journal.pcbi.1010076)
Supplement: Attachment — Submitted filename: report-PlosCompBio-SpatialOncolyticVirotherapy.pdf [file pcbi.1010076.s013.pdf]

Report on Manuscript PCOMPBIOL-D-22-00532:

Modeliing the spatial dynamics of oncolytic virotherapy in the presence of virus-resistant cells

by Darshal K. Bhatt, Thijs Janzen, Toos Daemen, Franz J. Weissing

Topic. This article studies a spatial agent based model of oncolytic virotherapy assuming that tumor cells may become resistant to the virus. For 3 types of spatial structure (2D regular, 2D voronoi, 3D regular), starting from a reference scenario, a number of parameters are varied in order to study their effect on treatment outcome. These parameters are: rate of viral spread, death rate of infected cells, time of introduction of the virus, sensitivity of stromal cells to the virus, possibility or not to infect cells that are not immediate neighbors. The possible treatment outcomes considered are: Total cancer eradication partial cancer eradication, sensitive cancer persistence, resistant cancer persistence.

Novelty. I did not check the literature but the authors write that, among models on oncolytic virotherapy, this is the first spatial model with tumor virus-resistant tumor cells.

Soundness. The article seems scientifically sound.

Writing. The writing is clear, easy to follow.

Figures. Figures are well done, pedagogical.

Choice of parameters. I am not an expert of oncolytic virotherapy and I do not feel competent to judge whether the model basic parameters are plausible or not.

Overall impression. I think that the article is well written, serious, and does what can be expected of an Agent-Based-Model, that is, explore and visualize the impact of various parameters on treatment outcome. The authors sometimes manage to provide some intuitions for the result of their simulations, which is good, and sometimes not, which is frustrating, but often the case with ABM models. There are of course many other directions that could be explored but one has to stop somewhere. I did not detect claims that seem too bold. Let me just mention that the discussion insists on the idea of sensitizing stromal cells to virus infection, though line 252-255 mentions that the parameter range where the virus sensitivity of stromal cells is beneficial is relatively small and that this virus sensitivity also has a cost. So maybe the discussion could be toned down on this aspect. Other than that, I make a few remarks below but more as food for thought, not to require any particular changes.

Miscellaneous remarks

The remarks below are not meant to lead to any change in the article, this is just food for thought. No reply is necessary.

- l. 129: I wonder whether a difference between infected cancer cells and infected stromal cells would make sense.
- l. 137-140: you might mention here that $S_s < 1$. Moreover, the sentence "A virus-infected cell infects a neighboring node with a probability [equal to] the susceptibility of this node, where the susceptibility of a susceptible cancer cell is given by 1" confused me. First, I think "probability" should be replaced by "rate", second the default rate of viral spread b_i in Table 1 is not 1 but 1.2.
- I understand from Table 1 that infected cells never proliferate, is this written somewhere?
- Here are some variants: partially resistant tumor cells (as opposed to all or nothing), partially sensitive stromal cells, backmutations (or phenotypic switching), possibility of infected cells to proliferate (not sure this is realistic), cell motility,...
- l. 212-213 and many figures: about the ratio d_i/b_i, or rather its inverse. It seems to me that the quantity $1/d_i$ is more or less the expected number of periods in which an infected cells can contaminate other cells (I think exactly so if the ABM is such that whether the cell dies or not is resolved before it can infect another cell), and so the ratio b_i/d_i is related to the number of cells an infected cell would contaminate if always fully surrounded by sensitive cells. The fact that this ratio seems to have a relatively meaningful interpretation may help to interpret some of the figures where, at least sometimes, the curves separating two domains are close to straight lines, that is, roughly correspond to a constant value of the ratio b_i/d_i.
- How likely is it that at least some resistant tumor cells exist at the time of introduction of the virus? (and is this quantity important, or not so much).
- Impact of cell number and cost of resistance: if a much larger number of tumor cells were considered, it would be very likely that at least some resistant tumor cells appear early on. However, if there is a cost of resistance, these cells would be outcompeted by sensitive cells before the introduction of the virus, so maybe the situation would not be that different than with a small number of tumor cells. If there is no cost of resistance, and if whether resistant cells are already present or not at the time of virus introduction is an important factor (not sure), then I would expect different results when simulation a much larger tumor (which seems more realistic).
- What is the impact of 2D versus 3D in the formation of barriers? I would expect barriers are more common in 2D.

- l. 343-346: how is the range from 10^{-6} to 10^{-2} for the rate of occurence of resistance per cell division connected to the finding that there are at least $1-10\%$ of resistant cells in patient-derived cancer cell-lines (and is this before or after introduction of the virus)? Is there a rough math formula? What is the proportion of resistant cells at the time of virus introduction that you find in your simulations? I would also expect the resistance cost to impact the initial proportion of resistant cells.

- l. 369-371: failure during early therapy vs failure during late therapy. Can this finding be used to stratify patients in any way? To have different virotherapies depending on initial tumor size or other factors?

- l. 384-387, impact of grid size: my understanding is that the grid size will not affect the probability of having already resistant cells at the start of therapy (because anyway the tumor would not yet have invaded the whole grid) but may become important later on.

- l. 392-393 (see also l. 405-406): the fact that in 3D, cancer cells were able to persist more than in 2D is somehow at odd with the fact that one of the mechanisms of persistance is the appearance of barriers, and that barriers should be harder to form in 3D (intuitively).

- l. 412: "a degree of sensitivity to viral infection": of which cells?

- Figure 2: comparing rows A and D, it is not obvious that in row D, treatment failure is due to resistant cells, it also seems that, somewhat stochastically, some sensitive cells (far from the resistant cells cluster) failed to be infected and then repopulated the tumor.

- l. 481: comma missing.

- Supplementary Figure 3: there are more similarities between the three rows in panel A than in panel B.

- Supplementary Figure 6, panel A, right (high mutation rates): this could look different if backmutations (or phenotypic switching) were considered.
