# Supplementary material for: Modelling the spatial dynamics of oncolytic virotherapy in the presence of virus-resistant tumour cells
Source: PLoS Comput Biol. 2022 Dec 6;18(12):e1010076. doi: 10.1371/journal.pcbi.1010076 (PMC9767357; doi:10.1371/journal.pcbi.1010076)
Supplement: Attachment — Submitted filename: response to reviewers.docx [file pcbi.1010076.s014.docx]

**Response to reviewers**

We would first like to thank the reviewers for providing valuable feedback on our manuscript. In response to the reviewers’ comments, we have revised the manuscript considerably. In particular, we made the following changes:

- We clarified the model structure by adding a flow scheme to Fig. 1 (now Fig. 1E).
- We improved all data figures by making more explicit which parameters were varied and by stating more explicitly on which parameter setting the simulations shown were based.
- We expanded our analysis by adding a new model parameter (degree of resistance *R*_r_) and by exploring the effect of our model parameters on the outcome of oncolytic virotherapy more completely and more systematically. The results are shown in new figure panels (Fig. 4B, Fig. 6C) and new supplementary figures (Fig. S2, Fig. S3, Fig. S8D, Fig. S10). Moreover, we changed the setup of Fig. 5.
- We added a new supplementary figure (Fig. S1) to allow visual comparison between the Voronoi model and the 3D model run with the same parameters.
- We improved the model description at various places (e.g. the implementation of the Gillespie algorithm) and included additional references to models in virotherapy.
- We added some remarks and a reference about the mechanisms underlying resistance.
- We proofread the text and made corrections wherever required. As we incorporated virtually all textual suggestions of the reviewers, we will not discuss these suggestions in our responses below.

Below, the reviewers’ comments are reproduced in color while our response is given in black.

**Response to Reviewer #1:**

The article develops probabilistic, cell-based models to study the dynamics of anti-cancer virus therapy, known as oncolytic virotherapy. Their model considers four populations: normal healthy (stromal) cells, infected cells (cancerous or stromal), infection-sensitive cancer cells, and infection-resistant cancer cells.

Then, the authors conduct a systematic investigation of the model’s outcomes and how they depend on parameter values and other assumptions. They simulate three different types of cell-based models – 2D lattice, 3D lattice, and Voroni – and show that the results are qualitatively similar with a few variations.

A positive aspect of the work is that the authors develop and simulate three different types of models, 2D, 3D, and Voroni, which helps establish credibility that their results are not simply artifacts of the type of model.

Another positive aspect is that they conduct a systematic investigation of different scenarios and parameter combinations, such as the dependence of cancer eradication on the ratio of death rate of infected cells and viral spread rate; the influence of the timing of virotherapy; and influence of various parameters, e.g., production rate of resistant cancer cells, virus diffusion distance, and probability of virus infection. They also consider the possibility of allowing normal healthy cells to get infected and show that this scenario could increase the chance of complete cancer elimination, a successful outcome, but with the risk of increasing the population resistant cancer cells, leading to therapy failure.

We are of course pleased with these positive remarks on our manuscript.

The investigation could be even more systematic, such as by varying all model parameters and reporting variations and trends in outcomes as a table or histogram. This would make the study even stronger.

As indicated above, we have included an additional parameter in our model (degree of resistance *R*_r_) and more comprehensively and systematically explored the dependence of the therapeutic outcome on virtually all model parameters. This resulted in three new supplementary figures and three new figure panels. Reporting variations and trends in outcomes as a table is a good idea, but we decided against adding such a table and instead made the figures on parameter dependence more transparent.

The main weakness of the study is that the results do not seem innovative or novel. They seem very sensible and often expected. In addition, the treatment suggestion to consider an anti-cancer virus that can also infect normal healthy cells in an attempt to eliminate cancer is also not a very new idea. Consequently, this article does not provide the level of novelty or innovation suitable for PLoS Computational Biology.

We think that our study is novel in various ways. Our model is the first spatial model allowing the systematic investigation of how the presence of virus-resistant tumor cells affects the outcome of tumor virotherapy. The model allows the user to compare spatial configuration effects on the therapeutic outcome. Moreover, the model is encoded in a very efficient manner, allowing practitioners to run many simulations in a brief period of time on a standard pc.

It is true that many (but not all!) of the simulation results are in line with intuitive expectations. This, however, does not compromise the novelty of the underlying model. It is by no means self-evident whether the intricate interplay of virotherapy and viral resistance would lead to outcomes that have a seemingly straightforward interpretation. Only a model like ours can lead to a conclusion like this. Moreover, the model allows a more quantitative evaluation of the effects of the various model parameters. Last, but not least, the model also shows that in some parts of the parameter range the outcome of virotherapy is by no means deterministic, as different therapeutic outcomes co-occur under identical conditions.

Minor comments:

In line 138, it says “A virus-infected cell infects a neighboring node with a probability that is identical with the susceptibility of this node, where the susceptibility of a susceptible cancer cell is given by 1.” Is it a reasonable assumption that cancer cells get infected by infected neighbors with probability 1? It probably will not change the results much to vary this assumption, but the authors ought to mention the possibility that the infection probability is less than 1 and briefly note how that affects outcome without needing to show additional simulation results. It seems that the authors vary this assumption in Figure 6, but they ought to mention here that they will do it so that it does not seem as if they are ignoring this possibility until the reader comes to Figure 6.

We agree with the reviewer that the probability of infection via contact may be less than one. However, our model can already account for this by changing the rate of viral spread via contact. Reducing this rate by a certain factor has the same effect as reducing the probability of infection via contact by the same factor. We have now explained matters more clearly in **lines 157-162**.

We would like to clarify that **Figure 6** does *not* refer to the probability of infection via contact, but to the probability of a cell being infected at a distance.

Around Table 1, the authors ought to provide a parameter estimates subsection to discuss the parameters and their estimates. Summarize why the estimated values are the way they are even if the values came from Berg et al. or another paper.

We have now included the following explanation in the text (**lines 209-217**):

*“In view of the huge differences between tumours and types and designs of viral therapies, it is impossible to parameterize our model in a generally accepted manner. We therefore kept the default values of our parameters as similar as possible to those in the study of Berg et al. (2019), thus allowing a direct comparison of therapeutic outcomes in the absence (Berg et al. 2019) and presence (our study) of virus-resistant cancer cells. The parameter choice of Berg et al. (2019) was partly based on experimental data and partly on their aim to obtain the coexistence of stromal cells, cancer cells, and infected cells in different spatial configurations. In view of the latter consideration, the default parameter setting in our study may not represent the clinically most relevant situation. To compensate for that, we systematically studied a broad range of values for virtually all our model parameters.”*

Also, it seems parameter b_i for the rate of viral spread via contact of infected with susceptible cells is not discussed anywhere in the text. The authors ought to discuss this parameter in the text, too.

We have clarified the explanation of *b*_i_ in the manuscript (**lines 157-162**).

In Figure 4B,C,D, maybe the authors should plot the fraction of resistant cancer cells. Is it possible that the 3D model has a higher number of resistant cells just because it has more cells altogether? Is the proportion similar though?

In Figure 4, the total number of cells in the models is comparable in all three spatial configurations (10,000 cells in both 2D models and 10,648 cells in the 3D model). Therefore, the absolute number of resistant cells correlates with the fraction of resistant cells in all three configurations. This is explained in the manuscript (**line 146-148**):

*“By keeping the total number of lattice cells constant across the grid, we ensure that found differences between grids are due to their spatial organization rather than due to the size of the grid.”*

However, we agree with the reviewer that across spatial configurations the number of inhabited lattice cells might vary. We therefore replotted Figure 4 using the fraction of lattice cells in panels C, D, and E.

**Response to Reviewer #2:**

Overall feedback: In this manuscript, the authors develop a cell-based model of viral infection to investigate the dynamics of infection-resistance in tumour tissue. They compare three different variations of the model: two 2D models of tumour cell monolayers and a 3D model. They qualify how the therapeutic outcome is effected by properties of the virus, tumour and stromal cells as well as timing of treatment. Their simulations suggest three primary causes of therapy failure: rapid clearance of the virus, rapid selection of resistant cancer cells, and a low rate of viral spread due to the presence of infection-resistant healthy cells. Their model suggest that improved therapeutic efficacy can be achieved by sensitizing healthy stromal cells to infection. The results in this paper are very interesting and serve as a good study for the oncolytic virotherapy community. In places, however, I felt the assumptions of the model were not well supported or clarity around why certain avenues for investigation were ignored would be important to address. In turn, computational modelling of oncolytic virotherapy is a large field of research and I feel more justice could be done to reference previous models and their results in this area.

Major

• The authors introduce the notion of infection-resistant cancer cells impeding oncolytic virotherapy, however, this is not explained biologically and as such I feel gives rise to some confusion and questions. What are infection-resistant cancer cells? Why are they infection-resistant? Does the presence of infection-resistant cells depend on the virus, the genetic modification, and the underlying cancer line? Is the resistant due to a lack of conjugate receptor expression? Internal machinery needed for viral RNA replication? or is it resistance from IFN signalling? I believe it is very important that the authors outline what these infection-resistant cancer cells are to be sure that the readers can believe they exist as there is some debate in the field around infection-resistance cells in oncolytic virotherapy.

We thank the reviewer for this suggestion. A comprehensive review of the mechanisms underlying infection resistance is beyond the scope of this manuscript. However, we have recently published a systematic analysis of how oncolytic virotherapy is influenced by resistance mechanisms (Bhatt et al. 2021). We have now added some text on resistance mechanisms to the Introduction, and we also refer to our review paper (**lines 96-101**):

*“Resistance to virotherapy is a relatively unexplored area. Still, diverse mechanisms have been identified in the literature (reviewed in Bhatt et al. 2021), including interferon-mediated resistance, epigenetic modifications, hypoxia-mediated inhibition, APOBEC-mediated resistance, virus-entry barriers, and spatiotemporal restrictions to viral spread. In view of this diversity, we refrained from modelling a specific resistance mechanism and instead made more generic assumptions on the emergence of resistance.”*

• Why do the authors conserve the number of cell spaces from 2D to 3D? Is this a reasonable assumption and are they sure this doesn’t impact their predictions? Wouldn’t the tumour need the same amount of space to grow in a 2D plane as it would if you were just considering the 3D tumour? I think some clarity around this might help and maybe a figure of a simulation in the 2D models and the 3D models and comparing them would help alleviate my concern here (Something like Figure 2, but showing for the same parameter values what the three model predictions look like?).

• Along the same vein as the previous comment the authors state “Supplementary figure 1 shows that the simulation outcome is only marginally affected by grid size” but unless I am understanding Figure 1 incorrectly, increasing the grid size gives rise to larger and larger regions of the parameter space denoting resistance cancer cell persistence and less of the parameter space giving rise to sensitive cancer cell persistence and eradication. This needs to be clarified. Perhaps this might also be an explanation for why there is a large different in Figure 4 between the 3D model and two 2D models?

We have explained our choice regarding the grid size in the Methods section (**lines 141-148**). Our main interest was to understand whether and how the spatial organization of the tumour and the number of neighboring lattice cells affects our results. Conserving the number of lattice cells from 2D to 3D makes it easier to focus on the spatial configuration *per se*. We fully agree that the grid size has an effect on the outcome: in all three spatial configurations, the likelihood of the persistence of resistant cancer cells increases with the grid size. In the Discussion (now **lines 465-469**), this was explained by the fact that the generation of resistant cells is more likely in larger than in smaller grids. **Supplementary Figure 4** illustrates this effect of grid size, but it also reveals that the *differences* between the spatial configurations are only marginally affected by the grid size.

To be sure, there *are* differences between the 3D model and the two 2D models (for a given grid size), which are addressed in **lines 488-490** of the manuscript. We discuss various explanations, to which we have added the following:

*“We therefore hypothesize that the differences in simulation outcome are not caused by neighbour effects but by the fact that the degree of stochasticity is higher in the 3D model because of the extra degree of freedom.”*

We have added the new **Supplementary Figure 1** in order to visualize the concordances and differences between the dynamics in the 3D model and the Voronoi model.

• The authors state that “each node In the grid can be inhabited by one of four different cell types: healthy (stromal) cells, infected (cancer or stromal) cells..” shouldn’t infected cancer and infected stromal cells be considered differently? Surely infected cancer cells and infected stromal cells will have different properties for infecting neighbouring cells as they would replicate the virus differently? If the authors are assuming not then maybe this needs to be discussed and I wonder whether this assumption has any impact on the model predictions?

We agree that infected cancer and infected stromal cells will have different properties. However, they can at least partly be studied in our model. This is now explained in the manuscript in **lines 446-453**:

*“**The reader should notice that, for the sake of simplicity, our model assumes that infected stromal cells have the same rate of spreading the virus and the same death rate as infected cancer cells. This assumption will often not be realistic. However, a difference in the rate of virus spread (via contact) between the two types of cells can easily be achieved by changing the susceptibility of stromal cells to viral infection. When studying differences in the death rates of infected stromal and cancer cells (in an extended version of the model), we observed some potentially interesting patterns, which we will study in detail in future work.”*

• Can the authors discuss whether it is a limiting assumption to assume virus can only be transmitted from an infected cell to a neighbouring cell point? Viruses can diffuse through the tumour after secretion from infected cells and so in theory could diffuse past neighbouring resistant cells and infect susceptible cells. Did the authors confirm what impact it might have on the model if the radius of infection was increased to neighbours twice removed? I know they introduced the assumption that dead cells can transmit further, but I’m curious as to whether if the assumption for dead cells was given to infected cells what impact this might have on the model predictions?

It was one of our goals to produce a computationally highly efficient simulation model that allows practitioners to run many simulations in a brief period of time. For this reason, we refrained from incorporating virus diffusion from live infected cells in our model. The inclusion of such diffusion would have been computationally very costly, as it would have necessitated the calculation of viral spread through diffusion on a per-cell and per-timestep basis. This would have negated the efficiency of the Gillespie algorithm used in our model.

Although we did not implement the diffusion of viruses from infected cells prior to their death, our results on the effects of “infection at a distance” (upon the death of an infected cell) give a hint regarding the implications of viral diffusion. These effects are now summarized in the new **Figure 6C** and discussed in **lines 380-388**.

• It’s not surprising the authors did not find that infections in locations apart from the centre gave similar results as there was no spatial dependence on the likelihood of resistance, cell proliferation or viral replication. Could the authors comment on this? On whether the introduction of nutrient dependent (distance from edge) cell proliferation or incidence of infection-resistant mutation based on location to viral infection could impact the model predictions?

Our goal in comparing different locations of viral infection was to see if in the absence of *a priori* assumptions about spatial dependencies we would still find differences, driven only by the spatial location of infection. If so, this would suggest that the location of infection can affect the success of therapy. However, we did not find this. Rather, we found that the outcomes depend more on the rate of viral spread and infected cell death rate.

Indeed, we believe that introducing nutrient-dependent cell proliferation based on distance from the edge would impact the outcomes of our model. Considering the results from the new **Supplementary Figure 2**, we would hypothesize that a lower rate of viral spread in the core of the tumour will result in a higher likelihood of (resistant) tumour persistence. Similarly, considering the results from **Supplementary Figure 6**, we expect that a higher incidence of infection-resistant cells may critically impair the viral spread locally and lead to tumour persistence. However, it is also important to note that the persistence of resistant cancer cells is favored by the selective pressure provided by a viral infection in that local area, so it may be that resistant cancer cells are outcompeted (due to the cost of resistance) by susceptible cancer cells if the infected cells are absent in that area.

• Is it possible that the choice of tumour cell proliferation rate and other parameters like the death rate of healthy stromal cells could also be drivers of some of the sensitivity of the model predictions? Is it possible for the authors to comment on this or investigate this? It’s been shown that stromal density has an impact on OV efficacy, is this seen in your model? It might be part of varying stromal proliferation rate or density. In addition, could the authors elaborate on how one might make stromal cells “sensitive” to infection.

We have now systematically explored a whole range of values of the birth and death rates of stromal cells and (sensitive) cancer cells. The results can be found in the new **Supplementary Figures 2 and 3**. Indeed we observe that stromal cell density reduces the possibility of complete tumour eradication when the stromal cells are resistant to viral infection. On the other hand, complete tumour eradication also occurs if stromal cells outcompete cancer cells due to a relatively higher birth rate (*b*_s_) or lower death rate (*d*_s_). The results of the effects of cancer cell birth and death rates are explained in **lines 294-297**. Similarly, the results of the effect of stromal cell birth and death rates are explained in **lines 317-320**.

Stromal cells can be made ‘sensitive’ to infection by blocking antiviral signaling such as the JAK-STAT pathway, or by using oncolytic viruses that employ a non-specific mode of entry in the cell, or by modifying structural proteins of the virus to enhance binding to stromal cells. This has been tested experimentally and is referred to in the manuscript in **lines 437-439**.

Minor

• It feels like think there is a lack of review of the mathematical/computational literature surrounding agent-based models of oncolytic virotherapy, especially in the context of heterogeneous stromal tumours or Voronoi tessellations. Consider referencing the below

o <https://doi.org/10.3390/cancers13215314>

o doi: 10.1016/j.isci.2022.104395

o <https://doi.org/10.1016/j.jtbi.2019.110052>

These are indeed good suggestions. We now refer to these references in the text.

• The lattice configuration in Figure 1A is unclear to me, I can see a green cell in the centre which I believe represents an infected cell, but then just a red region around it? Is this red region meant to represent 4 cells? Can this image be fixed to actually denote the lattice, similar to what is shown in the Voronoi Tessellation where there are lines for each cell?

We have updated the visualization of the 2D regular grid model such that each lattice cell has a black border to have a clear idea of the cell boundaries. This is depicted in **Figure 1A**.

• The authors should be very careful with using the terminology of “cells” to mean biological cells and “cells” to mean lattice points as it does get confusing in places. For example in supplementary figure 1 when the authors say “(a) 500 cells; (b) 10,000 cells” etc, do they mean initial number of cells or total cell lattice points? Maybe changing to lattice cells or lattice points will help?

This is a very good point. To avoid confusion between “grid cells” (locations in the grid) and “biological cells”, the former are now systematically referred to as “lattice cells.”

• I found the description around S_s confusing. Is S_s between 0 and 1? Shouldn’t the susceptibility of stromal cells be less than or as great as cancer cells? How does this all relate to the rate b_i? Can the introduction of the infection probabilities be expanded upon and more detail given please (particularly making clear the assumptions around infection).

We have updated the explanation of the term “susceptibility of stromal cells (*S*_s_) towards infection” (**line 157-162** and **Table 1**). *S*_s_ is the probability that a contact-based viral transmission (attempt) actually results in viral infection. As a probability, *S*_s_ is in the range from 0 to 1. It is smaller than or equal to the susceptibility of cancer cells, which, for simplicity, we set equal to 1.

• Is Figure 2 the Voronoi 2D model or otherwise? Please state. What are the black regions in this figure? A suggestion would be to rotate Figure 2 90 degrees so we can see your animations clearer as they are very small currently.

Indeed Figure 2 displays the Voronoi model, where black regions indicate empty lattice cells.

• While the figures are lovely, I found the text on the majority of the figures way too small and difficult to read. Could the authors please consider fixing this for the readers?

We have updated the legend and axis labels in all the figures to improve readability.

• In Table 1 it says C_r is a production rate but in the text I believe it said it was a probability? Can this be clarified? Potentially the table needs a column saying the units so that it is clear what is a probability and what is a time-dependent rate?

Good point. *C*_r_ is a probability. This is now correctly explained in **lines 168-170** and in **Table 1**. Table 1 also indicates the units of all rate parameters.

• There is a difference between a “rate” and “probability” in the context of a cell-based model and the authors should elaborate on their explanation for the Gillespie algorithm. Consider adding a few steps of the algorithm to the supplementary information for reproducibility.

We agree with the reviewer that we did not clearly distinguish between probabilities and rates. To make this more explicit and clear, we have changed our wording in **Table 1**, added a graphical representation of our model to **Figure 1**. Moreover, we have expanded the description of the Gillespie algorithm in **lines 174-192** in the Methods section.

**Response to Reviewer #3:**

**Topic.** This article studies a spatial agent based model of oncolytic virotherapy assuming that tumor cells may become resistant to the virus. For 3 types of spatial structure (2D regular, 2D voronoi, 3D regular), starting from a reference scenario, a number of parameters are varied in order to study their effect on treatment outcome. These parameters are: rate of viral spread, death rate of infected cells, time of introduction of the virus, sensitivity of stromal cells to the virus, possibility or not to infect cells that are not immediate neighbors. The possible treatment outcomes considered are: Total cancer eradication partial cancer eradication, sensitive cancer persistence, resistant cancer persistence.

**Novelty.** I did not check the literature but the authors write that, among models on oncolytic virotherapy, this is the first spatial model with tumor virus-resistant tumor cells.

**Soundness.** The article seems scientifically sound.

**Writing.** The writing is clear, easy to follow.

**Figures.** Figures are well done, pedagogical.

**Choice of parameters.** I am not an expert of oncolytic virotherapy and I do not feel competent to judge whether the model basic parameters are plausible or not.

**Overall impression.** I think that the article is well written, serious, and does what can be expected of an Agent-Based-Model, that is, explore and visualize the impact of various parameters on treatment outcome. The authors sometimes manage to provide some intuitions for the result of their simulations, which is good, and sometimes not, which is frustrating, but often the case with ABM models. There are of course many other directions that could be explored but one has to stop somewhere. I did not detect claims that seem too bold. Let me just mention that the discussion insists on the idea of sensitizing stromal cells to virus infection, though line 252-255 mentions that the parameter range where the virus sensitivity of stromal cells is beneficial is relatively small and that this virus sensitivity also has a cost. So maybe the

discussion could be toned down on this aspect. Other than that, I make a few remarks below but more as food for thought, not to require any particular changes.

We thank the reviewer for the largely positive evaluation of our manuscript.

**Miscellaneous remarks**

The remarks below are not meant to lead to any change in the article, this is just food for thought. No reply is necessary.

• l. 129: I wonder whether a difference between infected cancer cells and infected stromal cells would make sense.

We have already discussed this point in our response to Reviewer #2. In many cases, infected cancer cells and infected stromal cells will indeed differ in their characteristics. For reasons of computational efficiency, we did not implement this in our model. However, as indicated above, differences between the two types of cells can be simulated by changing the parameter *S_s_* (susceptibility of stromal cells to virus infection).

• l. 137-140: you might mention here that Ss < 1. Moreover, the sentence ”A virus-infected cell infects a neighboring node with a probability [equal to] the susceptibility of this node, where the susceptibility of a susceptible cancer cell is given by 1” confused me. First, I think “probability” should be replaced by “rate”, second the default rate of viral spread bi in Table 1 is not 1 but 1.2.

We have already discussed this point in our response to Reviewer #2. We have updated our explanation in **lines 157-162** and **Table 1**.

• I understand from Table 1 that infected cells never proliferate, is this written somewhere?

This is true as explained in **lines 157-158**.

• Here are some variants: partially resistant tumor cells (as opposed to all or nothing), partially sensitive stromal cells, backmutations (or phenotypic switching), possibility of infected cells to proliferate (not sure this is realistic), cell motility,...

We think that the results obtained on testing the influence of back mutations would be similar to scenarios where resistant cancer cells arise at a lower frequency. Moreover, we agree that it would not be very realistic to allow infected cells to proliferate as the viruses are supposed to be lytic in nature. Although the possibility of cell motility is interesting, we find it to be beyond the scope of our manuscript and perhaps a focus of future work.

We have included (**Supplementary Figure 10**) the possibility of partially resistant tumour cells by introducing a new parameter: the degree of resistance (*R*_r_). This has also been addressed in our response to the comments made by Reviewer#1.

The model also has the possibility of partially sensitive stromal cells. As shown in **Supplementary Figure 8**, this influences the outcomes in the parameter range where stromal cells mediate resistance and tumour persistence. Chances of tumour eradication improve upon increasing the susceptibility of stromal cells to be infected.

• l. 212-213 and many figures: about the ratio di/bi, or rather its inverse. It seems to me that the quantity 1/di is more or less the expected number of periods in which an infected cells can contaminate other cells (I think exactly so if the ABM is such that whether the cell dies or not is resolved before it can infect another cell), and so the ratio bi/di is related to the number of cells an infected cell would contaminate if always fully surrounded by sensitive cells. The fact that this ratio seems to have a relatively meaningful interpretation may help to interpret some of the figures where, at least sometimes, the curves separating two domains are close to straight lines, that is, roughly correspond to a constant value of the ratio bi/di.

This is a good point, which we now address in the manuscript (**lines 280-282**).

• How likely is it that at least some resistant tumour cells exist at the time of introduction of the virus? (and is this quantity important, or not so much).

It is indeed possible that there are resistant cells at the time of virus introduction. However, their number will be small, as such cells are selected against in the absence of the virus. We therefore assumed that all resistant cells arise *de novo*.

• Impact of cell number and cost of resistance: if a much larger number of tumor cells were considered, it would be very likely that at least some resistant tumor cells appear early on. However, if there is a cost of resistance, these cells would be outcompeted by sensitive cells before the introduction of the virus, so maybe the situation would not be that different than with a small number of tumor cells. If there is no cost of resistance, and if whether resistant cells are already present or not at the time of virus introduction is an important factor (not sure), then I would expect different results when simulation a much larger tumor (which seems more realistic).

A similar point was also made by Reviewer#1. Please see our response to the effect of grid size on the likelihood of the various therapeutic outcomes.

• What is the impact of 2D versus 3D in the formation of barriers? I would expect barriers are more common in 2D.

Indeed the formation of barriers seems to be less common in 3D, although it is difficult to visually assess this, due to the more complex nature of the spatial organization. Because lattice cells are more connected to each other, it is easier for cells to bypass each other and circumvent local barriers.

• l. 343-346: how is the range from 10−6 to 10−2 for the rate of occurence of resistance per cell division connected to the finding that there are at least 1 − 10% of resistant cells in patient-derived cancer cell-lines (and is this before or after introduction of the virus)? Is there a rough math formula? What is the proportion of resistant cells at the time of virus introduction that you find in your simulations? I would also expect the resistance cost to impact the initial proportion of resistant cells.

There is no indication so far as to how many resistant cells one can expect in the tumour, as it depends on the complex interplay of occurrence of resistance and the birth rates and death rates of stromal, resistant- and susceptible- cancer cells. The finding that there are at least 1-10% of resistant cells in patient-derived cancer cell lines is made after virus introduction. However, these numbers do not represent the occurrence of resistant cells *in vivo* as cell lines undergo clonal selection for optimal use in the laboratory.

• l. 369-371: failure during early therapy vs failure during late therapy. Can this finding be used to stratify patients in any way? To have different virotherapies depending on initial tumor size or other factors?

Interesting point. But we would need more information to make this possible.

• l. 384-387, impact of grid size: my understanding is that the grid size will not affect the probability of having already resistant cells at the start of therapy (because anyway the tumor would not yet have invaded the whole grid) but may become important later on.

Yes this is true.

• l. 392-393 (see also l. 405-406): the fact that in 3D, cancer cells were able to persist more than in 2D is somehow at odd with the fact that one of the mechanisms of persistance is the appearance of barriers, and that barriers should be harder to form in 3D (intuitively).

Upon introduction of cancer cells, these cells are placed in the centre of the grid, forming either a circle (2D), or a sphere (3D). Expansion or growth of the cancer cells can only occur at the edge of the tumour, which is relatively bigger in 3D than in 2D. As a result, typically, we observe that in 3D there is a bigger initial expansion of cancer cells, which contributes to their persistence. This seems to be independent of the formation of barriers. Yet, we agree with the reviewer that it would be very interesting for future work to identify the formation of spatial barriers and analyze how this relates to spatial configuration.

• Figure 2: comparing rows A and D, it is not obvious that in row D, treatment failure is due to resistant cells, it also seems that, somewhat stochastically, some sensitive cells (far from the resistant cells cluster) failed to be infected and then repopulated the tumor.

This is true. We picked another example where the effect of resistant cells causing therapy failure is clear.

• Supplementary Figure 3: there are more similarities between the three rows in panel A than in panel B.

The discrepancies may be explained due to the differences in model design, and the observations here were indeed meant to demonstrate similarities.

• Supplementary Figure 6, panel A, right (high mutation rates): this could look different if backmutations (or phenotypic switching) were considered.

We think that the results obtained on testing the influence of back mutations would be similar to scenarios where resistant cancer cells arise at a lower frequency (**Figure 4A**) or have a lower degree of resistance (**Figure 4B**).

We would like to thank all three reviewers again. We hope (and have the impression) that their comments and suggestions have improved our manuscript considerably.
